# Experiencing Positive Health, as a Family, While Living With a Rare Complex Disease: Bringing Participatory Medicine Through Collaborative Decision Making Into the Real World

**DOI:** 10.2196/17602

**Published:** 2020-06-22

**Authors:** Caridi Family, Annapurna Poduri, Orrin Devinsky, Miriam Tabacinic, Alejandro R Jadad

**Affiliations:** 1 The Caridi Family Aventura, FL United States; 2 Epilepsy & Clinical Neurophysiology Boston Children's Hospital Harvard University Boston, MA United States; 3 Comprehensive Epilepsy Center and the Saint Barnabas Institute of Neurology and Neurosurgery New York University New York, NY United States; 4 T Design Miami, FL United States; 5 Dalla Lana School of Public Health University of Toronto Toronto, ON Canada

**Keywords:** collaboration, shared decision making, patient–physician relationship, communication, partnership, participatory medicine

## Abstract

Physician–patient collaboration was recognized as a critical core of participatory medicine more than a century ago. However, the subsequent focus on scientific research to enable cures and increased dominance of physicians in health care subordinated patients to a passive role. This paternalistic model weakened in the past 50 years—as women, minorities, and the disabled achieved greater rights, and as incurable chronic diseases and unrelieved pain disorders became more prevalent—promoting a more equitable role for physicians and patients. By 2000, a *shared decision-making model* became the pinnacle for clinical decisions, despite a dearth of data on health outcomes, or the model’s reliance on single patient or solo practitioner studies, or evidence that no single model could fit all clinical situations. We report about a young woman with intractable epilepsy due to a congenital brain malformation whose family and medical specialists used a *collaborative decision-making approach*. This model positioned the health professionals as supporters of the proactive family, and enabled them all to explore and co-create knowledge beyond the clinical realm. Together, they involved other members of the community in the decisions, while harnessing diverse relationships to allow all family members to achieve positive levels of health, despite the resistance of the seizures to medical treatment and the incurable nature of the underlying disease.

## Introduction

Collaboration between physicians and patients in a mutual relationship—the core attribute of participatory medicine—was first documented in the late 19th century [1]. Bertha Pappenheim (also known as Anna O.) and Dr Josef Breuer, her physician and Freud’s mentor, discovered the therapeutic power of a collaborative partnership in the 1880s while listening to and learning from each other [2]. In the late 1950s, the *mutual participation model* was relegated to psychoanalysis and psychology. Traditional medicine favored models that had existed since Ancient Egypt, which placed physicians as the dominant members of the relationship, and patients mostly as passive or inactive beings. These *paternalistic*, *priestly*, or *passive* models [3-5] pervaded 20th century medicine, driven by a hierarchical view of knowledge, with physicians at the apex and patients/families at the base, a reductionist *chemical–mechanical* view of people, a pathophysiological approach to illness, and a belief that science could conquer disease and even defeat death [6].

As most acute conditions (eg, infections, diabetic coma, appendicitis) became curable, chronic incurable diseases dominated, leading to models based on *doing things to patients* [7]. This approach to chronic disease is rife with limitations, and in some cases (eg, chronic pain and psychiatric disorders), modern medical approaches may do more harm than good. By the early 21st century, a *shared decision-making model* had gained ascendance as *the pinnacle* for clinical decisions, particularly within the context of evidence-based medicine and patient-centered care [8-11]. Even though there are multiple ways to conceptualize it, in essence, shared decision making incorporates at least two participants—typically 1 physician and 1 patient—who examine information about different options to manage a condition, taking steps to build a consensus and to agree about which one to implement [12].

The shared decision-making model echoes and builds on precursors from the mid-1900s, which failed to cross from theory into clinical practice. Current approaches reflect the reaction against paternalism in the physician–patient relationship. They received names such as *mutual participation* or *collegial* models [3,7], and were also regarded as alternatives to another model, which considers physicians as the main source of facts and synthetic advice, for patients to weigh relative values and make the diagnostic or therapeutic decisions. These have been labeled *consumerist*, *informative*, *informed*, *autonomous*, or *engineering* models [3,7,13-16]. Despite being touted as the ultimate model, several systematic reviews of shared decision making reveal a dearth of supportive evidence, leaving their impact on empirical health outcomes uncertain [10,17-23].

Others observed that most research on shared decision making does not match clinical reality, because studies focus on a single patient with a solo practitioner. Instead, the real-life situations employ the model with patients who do not want to make decisions alone, preferring their loved ones to be involved or take charge in making critical decisions, and with multiple specialists participating in their care [24]. Besides, patient preferences for a shared decision-making model vary across studies according to their date of completion, as well as the selected population and the measurement tools used [25]. Further, the sheer diversity of models of relationship might indicate that the needs and preferences of patients and clinicians differ, and that existing models are components of a menu from which to choose, rather than single, fixed options to use during their interactions [26].

Our case illustrates how a new model, *collaborative decision making*, enabled a family and a group of involved health professionals to overcome all of the aforesaid limitations. This new approach, which was proposed in this journal in 2010 as an invitation to those involved in participatory medicine to consider a shift from the *shared* model, is presented here as an option to enrich, rather than to replace or displace, all other options, as it could foster a stronger partnership among patients, loved ones, and professionals, encouraging them to engage in a process with the common goal of creating a plan of action aimed at improving health [27].

The description follows the parameters that reflect the range of interests of those involved in participatory medicine, and underscores the desire of an entire family that leveraged this model to find solutions not offered by leading institutions, and to bring their experiences to other patients and health professionals who could learn from it.

## Case Presentation

### The People

Silvana was a 14-year-old woman when she was diagnosed with subcortical band heterotopia in 2011, following a seizure during a flight. This rare condition results from millions of neurons that do not migrate properly during development, creating a brain with dense bands below the cerebral cortex, where there should only be white matter fibers connecting neurons [28]. This explained the mild learning impairment that Silvana experienced throughout her life and the drug-resistant focal epilepsy that was progressively worsening for 5 years. She had 4 different seizures types, occurring at least once per week each but some up to 30/day, and lasting 4-150 seconds. Typical seizures included a fixed stare, shaking, or trembling of one hand, without loss of awareness, followed by fatigue. Every few months, she would have a drop episode. Despite these challenges, Silvana remained a cheerful young woman, keen to be offered tasks to complete, and eager to engage in artistic pursuits, especially photography and painting. Her main concern was, consistently, not to be left alone in an enclosed environment, such as an elevator, because of her fear of injury as a result of a fall.

The severity of Silvana’s condition disrupted her family’s life, with her father Ricardo most affected. He was frustrated by the trial-and-error approach to the frequent changes in the dose and combination of antiseizure medications by the multiple physicians involved in her case, despite understanding the dearth of scientific evidence supporting any option over the others. Using the skills and attitudes that had enabled him to become a successful entrepreneur, such frustration was transformed into a relentless urge to become an expert on band heterotopias and to perform online searches, almost compulsively, seeking to find a silver bullet that could have been missed by all of the specialists involved in his daughter’s care. He also joined groups of parents on social media, hoping to find and benefit from additional insights from the field. The frustration associated with the failure to find an effective treatment for the seizures morphed into exhaustion and anxiety so intense that he required support from psychologists and psychiatrists, with little benefit. An additional source of distress was the regret produced by the realization that he would feel much better whenever Silvana was out of his sight, especially in a different city.

Silvana’s mother, Denise, faced different challenges. She accepted the problem’s incurable nature and its complexity, which meant no doctor had sufficient data to guide therapy accurately, and sought to reduce its social impact. She fought the stigmatization by the family’s relatives and friends as well as potential rejection by peers, and the interruption of Silvana’s high-school studies. Given the conservative city in which they lived, the family decided not to disclose the underlying neurological condition to the school until the last year of studies, or to people outside the inner family circle. Instead, Silvana was diagnosed with a learning impairment.

Leon, Silvana’s only sibling, became very supportive of his sister, while maintaining his high academic level of school performance. Even as a young child accompanying his sister to medical visits, he was able to ask pertinent questions about *why* this happened to his sister’s brain, underscoring that the common sense questions of a child are among the most important questions that physicians should try to answer.

### New Knowledge Creation Through Collaboration of Researchers and Patients, as Individuals and as Groups

By 2016, Ricardo’s relentless efforts to find effective medicines to control Silvana’s seizures proved fruitless. As this was making his distress overwhelming, a physician friend of the family (Miriam Tabacinic), who was aware of the collaborative decision-making model, suggested to contact its lead author (Alex Jadad), whom she knew since their postgraduate training years back in the 1980s.

Given that they were located in different regions of the continent, Ricardo and Denise held an initial virtual conversation with Alex, during which the latter explained the model, emphasizing the need for a shift from a focus on the fight against the disease to one devoted to the enjoyment of health, and from Silvana to Ricardo as the person most in need of support.

Ricardo’s level of distress was extremely high and reducing it became the first priority. Because of his compulsive desire to cure Silvana’s seizures and the large swaths of time he was spending searching the biomedical literature and consulting specialists in different regions of the world, it was agreed that a formal synthesis of the literature on treatment-resistant epilepsy would be conducted, and that the leading authors would be invited to join a panel to discuss Silvana’s case and the best course of action.

The synthesis, which included papers indexed by MEDLINE and EMBASE from January 2015 to June 2016, was complemented by screening of all of the citations of relevant articles, and a forward search, using Google Scholar.

This systematically individualized effort to search, screen, and distill the peer-reviewed and gray literature revealed many options with a high probability of success still available to Silvana, including cannabidiol and other cannabinoids; conventional, first-line antiepileptic drugs; ketogenic or modified Atkins diets; noninvasive neurostimulation methods; experimental drugs; vagus nerve stimulation; or corpus callosotomy.

### Health Professionals and Health-Related Institutions

The relevant articles identified potential experts who were invited to become panel members to hold an in-depth discussion about which options to pursue (see Acknowledgments). They included Orrin Devinsky (Panel Chair, who also was Silvana’s current treating physician) and Annapurna (Ann) Poduri, another physician in Silvana’s team who managed her as a teenager and was highly trusted by her family.

The group acknowledged that a diverse panel of experts provided an opportunity for new insights to emerge to maximize Silvana’s health, while reassuring Ricardo and the family about the robustness of the recommendations. The panel held one whole-group session, chaired by Orrin, using a digital videoconferencing platform, which was followed by on-demand ad hoc email exchanges. After multiple interactions, invasive options were unacceptable to the family. Instead, it was decided to try a modified Atkins diet coupled with different combinations of conventional pharmacological interventions, leaving transcranial stimulation and experimental drugs to be considered at a later stage.

As Silvana was now an adult, it was agreed that Orrin would act as the main treating physician, working closely with Ann, to ensure continuity of care.

### Contextual Determinants

The rigor of the panel, the commitment of its members, and the open and comprehensive way in which Ricardo’s questions were addressed enabled a major shift. The family’s near-exclusive focus on the illness and its symptoms was broadened and redirected to a more constructive emphasis on health, acknowledging that it is much more than the absence of disease. They accepted a conceptualization that considers health as the ability of individuals and communities to adapt and manage the physical, mental, or social challenges faced throughout life [29]. This facilitated a much more effective and natural alignment between the family’s goals, the views of the experts, and the collaborative decision-making model to develop *an optimal action plan to improve health* [27].

This shift to a health-focused approach to Silvana’s life with intractable seizures enabled a transition from finding a cure or complete seizure control to achieving maximum levels of adaptation and self-management of an incurable condition through the activities illustrated in Table 1. Throughout the process, they agreed to monitor their levels of self-reported health by asking themselves the following question: “In general, would you say that your health is poor, fair, good, very good, or excellent?” Answering *poor* or *fair* represented negative health, whereas *good*, *very good*, or *excellent* was regarded as reflecting positive health.

Initially, Ricardo’s self-reports were consistently negative; Silvana’s and Leon’s were consistently positive; and Denise’s fluctuated, depending on whether she spent more time with Ricardo or their children.

**Figure 1:**
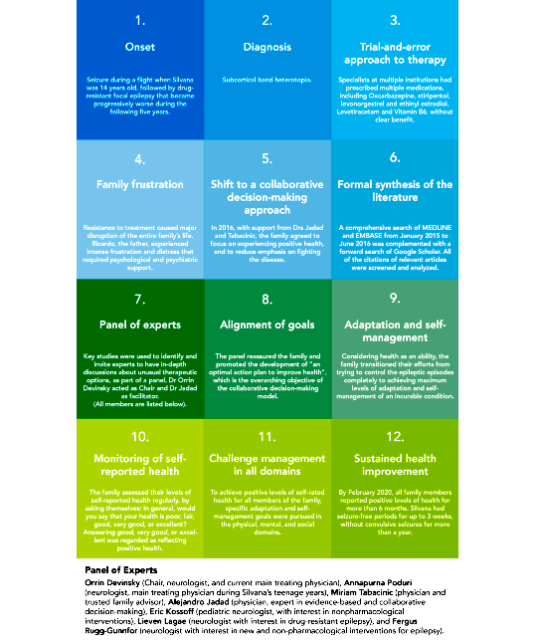
Key stages of the collaborative decision-making process within the context of participatory medicine.

The intention was to achieve positive health self-ratings for all family members for at least six months, to consider the approach successful and worth sharing with other families and health professionals. Figure 1 summarizes the journey.

**Table 1:** Efforts to generate maximum levels of adaptation and self-management within the context of health-focused collaborative decision making

Goals	Health domains		
	Physical	Mental	Social
Adaptation	Companionship in enclosed spaces and outdoors to prevent injuries	Recognition of the incurable nature of the disease	Adapted high-school curriculum On-site tutor to assist with academic tasks
Self-management	Optimal adherence to medication intake	Family-focused counseling, yoga, and meditation	Initiation of a small business with a close family friend

From the physical perspective, the diet added little value in seizure control despite sustained strict adherence, and medication reduced seizure frequency and duration without eliminating them. Therefore, the collective objective switched to reducing bodily injuries while more drugs were tried following a systematic trial-and-error approach.

From the mental point of view, the panel contributed significantly to Ricardo’s acceptance that Silvana’s condition was incurable and that she was receiving the best available therapies. Subsequently, the family engaged in individual yoga and meditation training, and group counseling to identify and set boundaries, which facilitated their collective adaptation to living with an intractable chronic condition. Silvana was encouraged and supported to build self-confidence, and to develop new ways to manage fears and strategies to respond to frustrating situations calmly. The entire family engaged in activities to reduce the reinforcing of Silvana’s sick role and to explore spiritual practices. The latter were particularly relevant for Ricardo, who decided to reconnect to his religious roots, finding solace and new sources of strength to deal with stress and to eradicate his guilt.

Socially, Silvana completed her high-school bilingual education with an adapted curriculum and support from an on-site tutor. Based on Silvana’s enjoyment of tasks requiring attention to detail, her family supported the creation of a small business focused on the manufacture and commercialization of one-of-a-kind fashion accessories with a close family friend, which proved that it would be possible for her to make a living doing something she likes.

**Figure 2:**
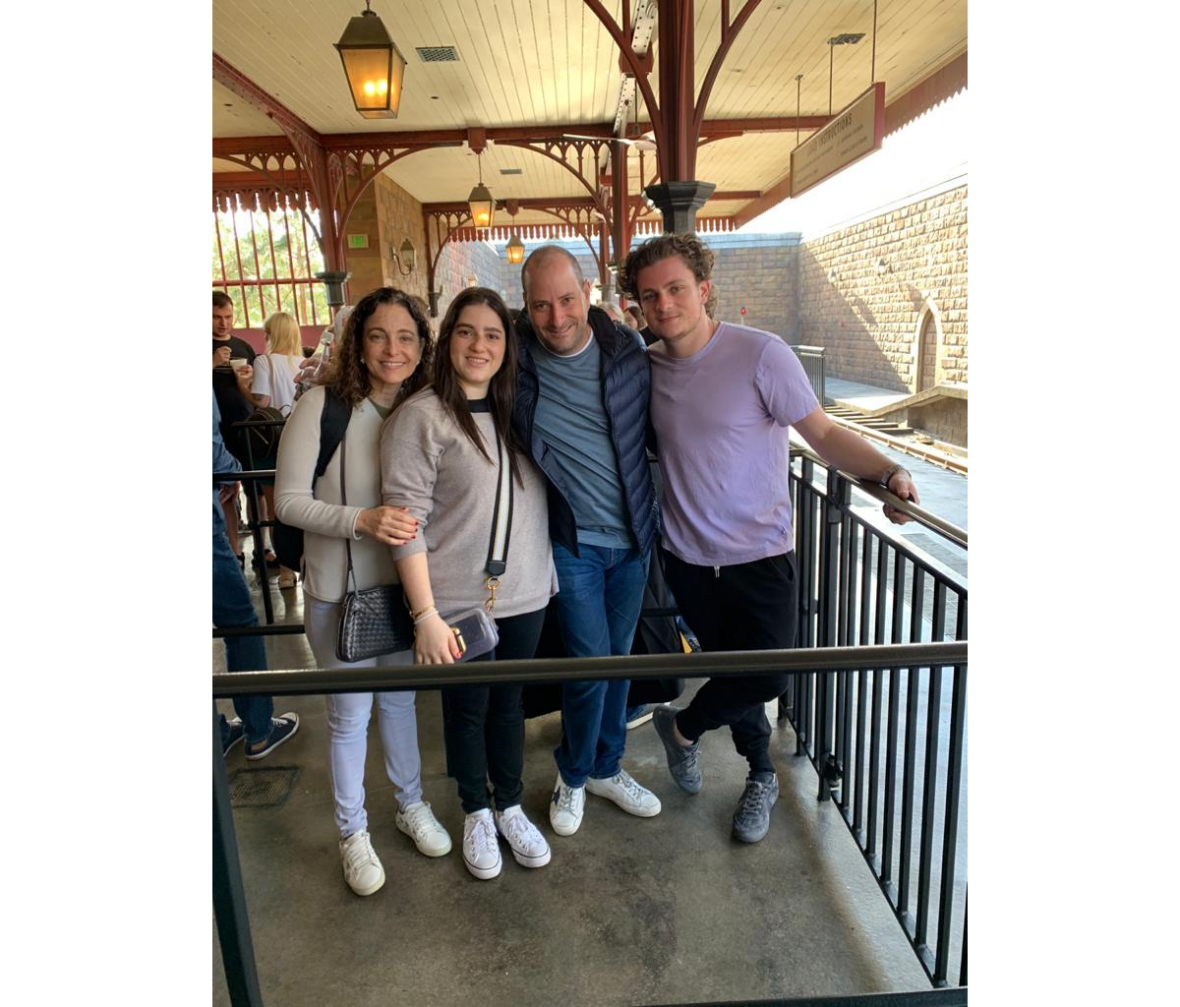
Denise, Silvana, Ricardo, and León Caridi in 2020.

Soon, all of these changes became part of their normal daily living, and their self-reported health status had stabilized at positive levels for all family members (Figure 2). Silvana summarized the situation by stating, “I feel better than I did before now that I have changed my habits. Yoga helps me concentrate on my breathing, and increases my awareness level. My family helps me by providing me with loving support and balance in my life.”

By February 2020, Silvana was taking lacosamide (400 mg/day), clobazam (40 mg/day), and vigabatrin (1750 mg/day), enjoying a significant and sustained reduction in her seizures. The most severe seizures caused head drop with impaired awareness for up to 30 seconds. She could go for up to 3 weeks seizure free. Anxiety and stress remained triggers, and when she reduced her daily yoga activity, her seizures would worsen.

By the time this article was submitted, Silvana was working with a cousin in her photography and graphic design company, designing a family cookbook. At that point, she had been free of convulsive seizures for more than a year.

## Discussion

This case illustrates how collaborative decision making could enable a family and their health professionals to use different relationship models, to explore therapeutic options within the broad domain of traditional and nontraditional medicine. The panel enabled Ricardo to benefit from the informative model by asking the participating specialists to answer his questions until he was reassured that the family’s decisions were based on the best knowledge. Once this was achieved, the panel promoted two-way knowledge exchange, shifting to a shared decision-making model, and focused on identifying the best course of action. Once there was agreement around the best course to follow, the family comfortably switched to a slightly paternalistic relationship, especially for diet implementation and systematic medication changes.

Conceptualizing health as the ability to adapt and self-manage the physical, mental, and social challenges created by the subcortical band heterotopias added value in fundamental ways. First, the family shifted years of emphasis on the disease to concentrating on achieving optimal levels of health per se. Part of this process was giving themselves permission to deviate from the *cure at all cost* paradigm. Second, this conceptualization helped the family recognize that all of them—not just Silvana—needed support to enhance their capacity to adapt to her intractable epilepsy. Lastly, it showcased how team effort can make positive health possible even in the presence of chronic, incurable diseases, opening new avenues for patients and clinicians to harness the power of collaboration as the essence of participatory medicine.
